# Evaluation of a Standardized Cardiac Athletic Screening for National Collegiate Athletic Association (NCAA) Athletes

**DOI:** 10.5811/westjem.2019.7.43190

**Published:** 2019-08-14

**Authors:** Chanel E. Fischetti, Reed W. Kamyszek, Stephen Shaheen, Benjamin Oshlag, Adam Banks, AJ Blood, Jeffrey R. Bytomski, Blake Boggess, Shadi Lahham

**Affiliations:** * University of California, Irvine Medical Center, Department of Emergency Medicine, Orange, California; † Duke University School of Medicine, Durham, North Carolina; ‡ Duke University Medical Center, Department of Surgery, Durham, North Carolina; § New York Presbyterian Hospital/Columbia University Medical Center, Department of Emergency Medicine, New York, New York; ¶ Duke University Medical Center, Department of Medicine, Durham, North Carolina; || Brigham and Women’s Hospital, Department of Medicine, Boston, Massachusetts; # Duke University Medical Center, Department of Community and Family Medicine, Durham, North Carolina

## Abstract

**Introduction:**

Sudden cardiac death is a rare cause of death in young athletes. Current screening techniques include history and physical exam (H and P), with or without an electrocardiogram (ECG). Adding point of care cardiac ultrasound has demonstrated benefits, but there is limited data about implementing this technology. We evaluated the feasibility of adding ultrasound to preparticipation screening for collegiate athletes.

**Methods:**

We prospectively enrolled 42 collegiate athletes randomly selected from several sports. All athletes were screened using a 14-point H and P based on 2014 American College of Cardiology (ACC) and American Heart Association (AHA) guidelines, ECG, and cardiac ultrasound.

**Results:**

We screened 11 female and 31 male athletes. On ultrasound, male athletes demonstrated significantly larger interventricular septal wall thickness (p = 0.002), posterior wall thickness (p <0.001) and aortic root breadth (p = 0.002) compared to females. Based on H and P and ECGs alone and a combination of H and P with ECG, no athletes demonstrated a positive screening for cardiac abnormalities. However, with combined H and P, ECG, and cardiac ultrasound, one athlete demonstrated positive findings.

**Conclusions:**

We believe that adding point of care ultrasound to the preparticipation exam of college athletes is feasible. This workflow may provide a model for athletic departments’ screening.

## INTRODUCTION

Sudden cardiac death is a rare but leading cause of death in young athletes on the playing field.^1^ These deaths are usually due to unsuspected heart disease, as many conditions are not detected by routine screening measures.^2^ National Collegiate Athletic Association (NCAA) athletes partake in rigorous training programs at an elite level. For collegiate athletes with a previously undiagnosed cardiac condition, the activity during training and competition places them at high risk for sudden cardiac death. The causes of sudden death in athletes under the age of 35 include hypertrophic cardiomyopathy (HCM), coronary artery anomalies, long QT syndrome, and infections such as myocarditis.

There are approximately 75 terminal outcomes per year in the United States in athletes between the ages of 13 and 25 years (89% occurring in males) with the majority immediately after exercise.^3^–^5^ New findings from an Italian Registry show a reduction of sudden death in athletes over the past decade due to enhanced screening of athletes, aged 16 and older.^6^ Pre-participation cardiovascular screening in athletes can uncover some of the underlying conditions contributing to this risk.^7^,^8^ The American Heart Association (AHA) and American College of Cardiology (ACC) Guidelines support screening with a 14-point history and physical examination (Appendix 1).^9^ However, studies have shown that the current screening techniques are insensitive in diagnosing many cardiac conditions.^7^,^8^ Early screening of patients at risk may improve the identification and early prevention of these cardiovascular events.^10^ Despite this data, there is still no universal and standardized applied screening method for incoming student athletes.^11^

A history and physical (H and P) examination without an electrocardiogram (ECG) are of questionable value and have not demonstrated cost-effectiveness due to their poor sensitivity and specificity.^12^,^13^ Prior studies have determined that routine screening with ECG and physical exam alone can detect some abnormalities.^14^–^18^ However, an issue posed by the AHA is the implication of medical liability in the current climate where no standardized means exist to clear student athletes for sport if they are deemed inappropriate to participate based on ECG findings.^11^ Other studies indicate that by providing a more standardized means for ECG analysis will provide a more homogenous and consistent interpretation of ECG screenings.^19^–^20^ In this study we aimed to assess the feasibility of conducting point of care cardiac ultrasounds in addition to routine preparticipation screening in collegiate athletes.

## METHODS

This study was approved by the site Clinical Review Board and the Institutional Review Board. Written, informed consent was obtained from all patients enrolled before any history, screening or ECG was completed. Our institution performs a standard 14-point ACC/AHA Pre-participation History and Physical Exam (PPE) and ECG on all incoming athletes during their freshman year. For this study, we offered a limited cardiac ultrasound exam as one additional component to the annual screenings.

### Subject Recruitment and Selection of Subjects

All male and female NCAA Division 1 student-athletes older than 18 years of age at our institution were invited to voluntarily participate during their intake collegiate athlete physical examination and cardiovascular screening visit. Potential subjects were recruited by convenience sampling. Athletes with previously known cardiac abnormalities were included in the study. Exclusion criteria included any athlete less than 18 years of age, all walk-on athletes (athletes not recruited or offered scholarship), or those not deemed part of the athletic program prior to the commencement of the academic year. Student athletes who did not agree to the study consent were excluded. Written consent was obtained from all athletes prior to participation.

**Population Health Research Capsule**

What do we already know about this issue?

*Sudden cardiac death is a rare cause of death in young athletes, usually due to unsuspected heart disease. However, there is still no standardized screening method*.

What was the research question?

*We assessed cardiac ultrasound in addition to routine preparticipation screening in collegiate athletes*.

What was the major finding of the study?

*Point-of-care ultrasound can be used to screen athletes for hypertrophic cardiomyopathy (HCM)*.

How does this improve population health?

*Future large-scale studies are needed to validate our promising findings and determine if ultrasound can be used as a screening tool for HCM*.

### Athlete Screening Workflow

All student-athletes completed their health history forms. A physical exam was then completed by one of the board-certified Sports Medicine physicians at the first station. At the second station, a trained ECG technician performed the ECG. During the process, a cardiology fellow was present and performed a preliminary read on the ECG. They were then read by an attending cardiologist using compiled ECG parameters from both the 2010 European Society of Cardiology Criteria and the Seattle Criteria (Refined Criteria) specific for athletes.^19^,^21^ Upon collection of the health history and physical exam information and ECG, the research team then performed a point of care cardiac ultrasound. All point of care ultrasounds were performed by trained emergency medicine resident physicians. These physicians received a 30-minute hands-on training session from the site ultrasound director. The data from these cardiac ultrasounds were then evaluated in real time by attending cardiologists with training in echocardiography. If abnormal findings on the ECG, physical examination or cardiac ultrasound were noted, these were immediately reviewed by one of the supervising cardiologists. If ECG or ultrasound abnormalities were confirmed, a full cardiac ultrasound and magnetic resonance imaging (MRI) were scheduled as a same week appointment with the Sports Cardiology clinic for further evaluation.

In summary, the overall workflow of athlete screening proceeded as follows:

1. Student athletes scheduled by the Athletic Department for their PPE, ECG, and point of care cardiac ultrasound exam.
2. Check in, voluntary Screening Registry introduction and informed consent provided to interested athletes.
3. Clinical visit with the physician to review 14 point AHA/ACC history and complete cardiac physical exam.
4. ECG is performed and reviewed (preliminary) by Sports Medicine or Cardiology Fellow.
5. Point of care cardiac ultrasound is performed and reviewed (preliminary) by Sports Medicine or Cardiology Fellow.
6. Abnormal ECG or cardiac ultrasound images are immediately shared and reviewed with attending Cardiologist.
7. All ECGs are reviewed by attending Cardiologist, then scanned into the athletes’ chart associated with their Cardiac Screen visit note.
8. For confirmed abnormal ECG or cardiac ultrasound, physical exam finding (heart murmur), or any other indication, a point of care cardiac ultrasound is arranged and a follow-up appointment (same week) is made with the Sports Cardiology clinic.
9. If no abnormalities are found (or confirmed), the student athlete may be cleared for the participation.
10. For student athletes who enter the athletic program at different times of year or have concerning cardiovascular events, the Sports Medicine faculty and Athletic Trainer perform an interim PPE and point of care ECG. These findings are immediately reviewed with the cardiology fellows and faculty on call. Abnormal ECG, cardiac ultrasound, physical exam finding, or event will lead to next day formal cardiac ultrasound and same week Sports Cardiology clinic visit. For more life-threatening events, athletes are transported to the nearest Emergency Department and Sports Cardiology fellow and faculty will be available by pager for stat consultation.

### Point of Care Cardiac Ultrasound Measurements

Based on American Society of Echocardiography guidelines, the inner left ventricular diameter breadth, interventricular septal wall thickness, posterior wall thickness, and the aortic root breadth were measured during diastole. All measurements were obtained using the parasternal long axis view with the patient lying in a supine position.^22^

#### Data Analysis

This registry did not duplicate the routine athletic screening process at our institution. All student athletes completed questionnaires during their screening session. Demographic data included gender, age, race and ethnicity, number of sports in which they compete, and specific sport was collected. Relevant family and personal cardiac health history were collected using the 14-point AHA/ACC history. Physical exam data was tabulated following the complete cardiac physical exam. The history and physical exams were recorded on paper and kept in the athlete’s permanent medical record prior to data entry in the registry. ECG and point of care cardiac ultrasound data were collected following interpretation by an attending cardiologist. All relevant data points were entered into a REDCap database. Male and female athletes were compared for differences in ECG and ultrasound measurements using unpaired Student’s t-test for continuous variables and Two-Proportion z-Test for categorical variables. A p-value <0.05 was considered statistically significant. All statistical analyses were performed using R statistical programming software version 3.4.2, (R Foundation for Statistical Computing, Vienna, Austria).

## RESULTS

**Figure 1:**
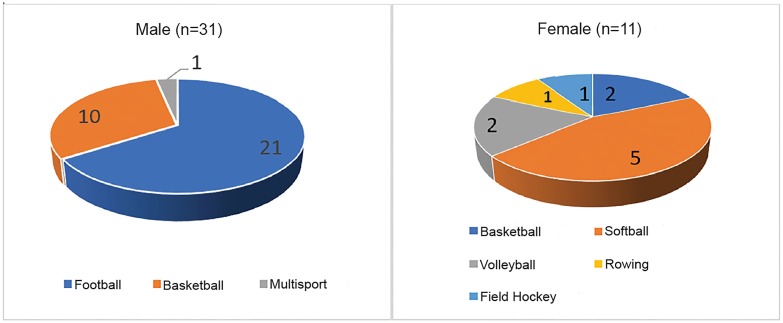
Screened athletes by sport.

50 athletes were approached for enrollment in the study. 42 athletes were screened with history and physicals from the available population in two days. The point of care ultrasound added approximately 7 minutes to each athlete’s screening. Most of this time was required for uploading and analyzing ultrasound images rather than image acquisition. The study group consisted of 11 female and 31 male athletes, with mean average ages of 18.5 and 18.6 years, respectively. Table 1 demonstrates demographics of all screened athletes. As shown in Figure 1, we screened 21 football, 10 male basketball, 2 female basketball, 5 softball, 2 female volleyball, 1 female rowing, and 1 female field hockey athletes.

H and P data demonstrated relatively benign family cardiac histories with collective family heart disease history prevalence less than 30% (Table 2). Personal cardiac histories most notably demonstrated a 12.9% prevalence of heart murmurs in males, and a 27.3% prevalence of syncope history in females. 19.4% of males and 9.1% of females reported a history of formal cardiac screening.

Overall, 41 of 42 athletes subsequently completed full ECG and ultrasound testing. We account for 41 of the 42 athletes on account that one of the athletes left before the ultrasound exam could be complete. Comparing male and female athletes, the two groups differed significantly in multiple ECG measurements. On average, males demonstrated longer QRS duration (98.2 milliseconds (ms) vs 90.0 ms, p = 0.004), and a higher proportion of athletes with J-point elevation (53.3% vs 18.2%, p = 0.044). Ultrasounds also demonstrated multiple significant differences. Males had significantly larger interventricular septal wall thickness (1.0 centimeter [cm] vs 0.8 cm, p = 0.002), posterior wall thickness (1.1 cm vs 0.8 cm, p <0.001) and aortic root breadth (2.7 cm vs 2.3 cm, p = 0.002) (Table 3).

**Figure 2:**
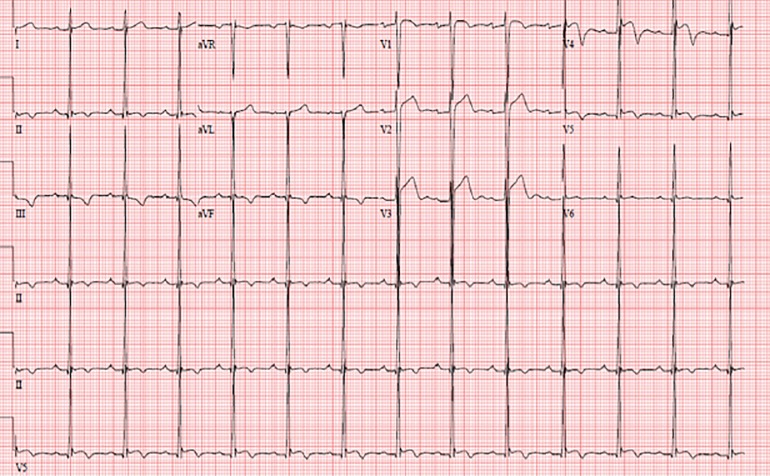
Electrocardiogram of athlete requiring follow up.

Based on H and P and ECGs separately, and when combining H and P with ECG, none of the 42 athletes demonstrated a positive screening for cardiac abnormalities. However, based on combined H and P, ECG, and point of care cardiac ultrasound data, one athlete demonstrated positive findings. This athlete was African American and demonstrated questionable findings in his H and P and ECG (Figure 2): he had a history of a heart murmur and notable ST elevations in his lateral leads (V1–5) with deep T wave inversions in II, III and V4, he also had a first-degree atrioventricular block.

**Figure 3:**
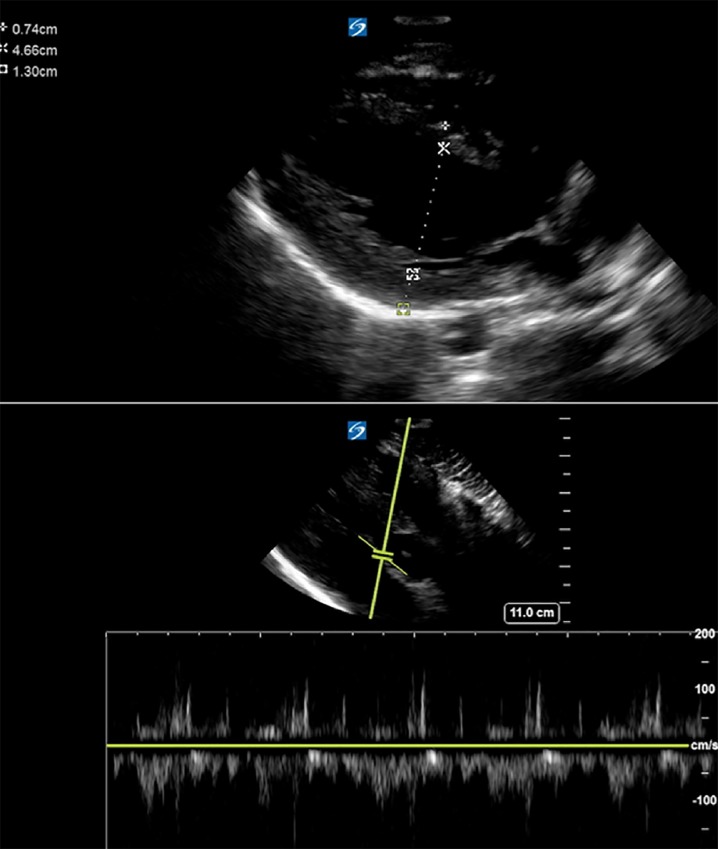
Ultrasound images of positive findings of point of care cardiac study. Posterior wall thickness of 1.3 centimeters coupled with the abnormal history and physical findings were concerning for this athlete.

**Figure 4:**
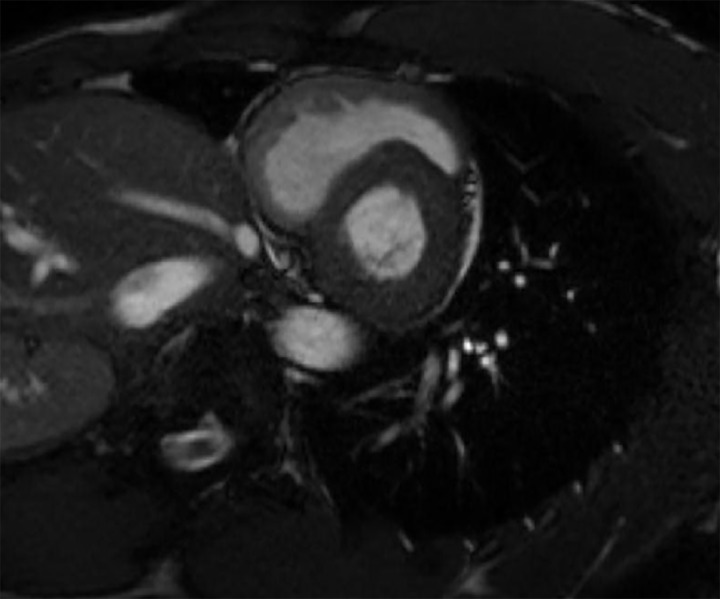
Still image from the cardiac magnetic resonance image of the positive athlete: Demonstrating concentric left ventricular hypertrophy with a septal thickness of 1.3 centimeters most consistent with an athletic heart.

These findings were consistent with refined Seattle criteria and would have warranted additional follow up. Coupled with his abnormal cardiac ultrasound findings, there was significant cause for concern, as he had an apparent enlarged left ventricle with a posterior wall diameter of about 1.3cm (Figure 3). Due to these concerning findings, he warranted additional imaging and follow up as an outpatient with cardiology and a cardiac MRI. The athlete’s follow-up cardiac MRI was evaluated as normal, although the athlete was found to have concentric left ventricular hypertrophy with a septal thickness of 1.3 cm most consistent with an athletic heart (Figure 4). Ultimately, the athlete was cleared for full participation.

## DISCUSSION

Many studies indicate that the history and physicals alone are poor representations of the actual assessed risk for pre-participation because of the low sensitivity and specificity of these findings.^12^ The ECG has been proposed as an inexpensive screening tool which may be added to the history and physical exam to identify athletes at risk.^10^ In fact, as mentioned by Harmon et al., the ECG can have important implications for primary prevention of sudden cardiac death. Estimates of the effectiveness of ECGs through screening alone range from 66% to 100%.^8^,^23^ Due to the low sensitivity of standard histories and variability of practices with ECG, many have proposed the addition of point of care cardiac ultrasound to routine screening procedures.

Although minimal significant cardiac abnormalities were identified in this study with the addition of point of care cardiac ultrasound, we were able to demonstrate efficiency in conducting pre-participation screening for athletes involving a comprehensive exam with point of care cardiac ultrasound. In our athlete population, we were able to obtain all four target ultrasound measurements in 100% of our athletes. As ultrasound becomes integrated into routine care models, it is reasonable to anticipate that non-cardiology trained physicians would be able to perform and interpret these exams.^24^,^25^

Based on our institution’s experience in coordinating this effort, this study is replicable if four key conditions are met: (1) an athlete’s individual screening occurs on a single-day; (2) the cardiac ultrasound creates minimal time disruptions to routine procedures; (3) ultrasound equipment is freely available for study team use; and (4) the presence of an attending cardiologist at the screenings is standard of care. First, due to our single-day screening format, all personnel, ultrasound equipment, and ECG equipment required for the study are preemptively coordinated to participate with minimal effort.

Logistically, all aspects of the study can be completed simultaneously due to this coordination of care teams and necessary equipment. Second, our athletic department policy dictates that all athletes obtain at minimum a H and P and ECG upon matriculation. Thus, the addition of a cardiac ultrasound, if kept to a minimum time requirement, is minimally disruptive to routine screening procedures. Third, available ultrasound equipment within a Sports Medicine department can help to minimize costs of this study and avoid logistical errors when obtaining a machine. Fourth, having a cardiologist present for the screenings is coordinated by our athletic department and set as standard of care. When considering the potential of using point of care bedside ultrasound as a screening technique, it is reasonable to consider using other trained providers to obtain these images, whether they are emergency medicine and ultrasound trained physicians or sonographers, the personnel can be varied. Thus, the task of coordinating an additional busy physician’s schedule to oversee the exam is mitigated. Thus, the preexisting standard of care for athletes at an institution is the largest facet to making this study, and addition of point of care cardiac ultrasound at any institution, feasible.

## LIMITATIONS

There are several limitations to this study. Patients were enrolled using a convenience sample and our data is therefore subject to sample bias. All athletes were unable to be represented in the study. Some were not available due to class interference, practice obligations, or leaving before research coordinator contact. Regarding medical history, information provided was limited to the participant’s knowledge of family and personal medical history. Family members with high risk histories were potentially omitted by student-athletes due to lack of knowledge or unwillingness to volunteer the information. We did not screen for any coronary artery abnormalities although this could be another cause of cardiac disease in young athletes. Our sample size was small and it is unclear if our findings can be generalized to the population. Future large-scale studies are needed to validate our findings. Based on the statistical prevalence of hypertrophic cardiomyopathy and other structural or congenital heart defects, this requires a much larger sample size to understand the utility of point of care cardiac ultrasound in detection of these conditions.

## CONCLUSION

Our study demonstrates the feasibility of a hypertrophic cardiomyopathy screening program that includes H and P, ECG and point of care ultrasound. We did not detect any cases of HCM in this small sample size. However, we believe that adding point of care ultrasound to the preparticipation exam is feasible. This workflow may provide a model for other athletic departments’ screening routines. This model could also serve cost-analysis studies for adding the ultrasound to routine protocols. Future large-scale studies are needed to validate our promising findings.

## Supplementary Information



## Figures and Tables

**Table 1:** Demographics of screened athletes.

Demographic	Male (n=31)	Female (n=11)
Age*, y	18.6 (18–22)	18.5 (17–22)
Hispanic/Latino, n	0	1
African American, n	21	2
Caucasian, n	10	9

* Mean (range)

**Table 2:** History and physical exam profiles of screened athletes.

	Male (n=31)	Female (n=11)
Physical exam findings		
Height* (centimeters)	190.8 (177.8–207.8)	175.8 (166.4–191.8)
Weight* (kilograms)	99.2 (73.8–153.4)	74.6 (62.1–88.5)
History findings**		
Family Hx heart disease	29.0	18.2
Family Hx hypertension	54.8	18.2
Family Hx unexplained syncope	3.2	0.0
Family Hx stroke	0.0	9.1
Family Hx pacemaker	0.0	9.1
Family Hx death < 50 years old	0.0	0.0
Family Hx structural heart disorder	0.0	0.0
Family Hx arrhythmia	0.0	0.0
Family Hx Marfan Syndrome	0.0	0.0
Personal Hx hypertension	0.0	0.0
Personal Hx heart murmur	12.9	9.1
Personal Hx formal cardiac screening	19.4	9.1
Personal Hx syncope during exercise	6.4	27.3
Personal Hx chest pain after exercise	3.2	9.1
Personal Hx pacemaker	0.0	0.0

*Hx*, history.

* Mean (range).

** History findings reported as % male or females screened.

**Table 3:** Electrocardiogram (ECG) and echocardiogram (ECHO) profiles of screened athletes.

ECG Data				

	Female (n=11)	Male (n=30)	All athletes (n=41)	p-value
HR (bpm)	63.4	65.4	64.9	0.644
PR (ms)	165.5	163.9	164.3	0.855
QRS (ms)	90.0	98.2	96.0	0.004
QT (ms)	397.1	396.3	396.5	0.941
QTc (ms)	404.7	408.4	407.4	0.690
Normal sinus rhythm	54.5% (6)	60.0% (18)	58.5% (24)	0.753
Sinus bradycardia	45.5% (5)	30.0% (9)	34.1% (14)	0.355
1° heart block	0.0% (0)	13.3% (4)	9.8% (4)	0.202
Axis deviation	18.2% (2)	10.0% (3)	12.2% (5)	0.478
J-Point elevation	18.2% (2)	53.3% (16)	43.9% (18)	0.044
T-Wave inversion	36.4% (4)	40.0% (12)	39.0% (16)	0.832
ST-Segment depression	0.0% (0)	3.3% (1)	2.4% (1)	0.540
ST-Segment elevation	0.0% (0)	16.7% (5)	12.2% (5)	0.149
Left atrial enlargement	0.0% (0)	10.0% (3)	7.3% (3)	0.276
Right atrial enlargement	9.1% (1)	10.0% (3)	9.8% (4)	0.931
Right ventricle hypertrophy	9.1% (1)	13.3% (4)	12.2% (5)	0.713
Complete LBBB	0% (0)	0% (0)	0% (0)	NA
Complete RBBB	0.0% (0)	3.3% (1)	2.4% (1)	0.540
Incomplete RBBB	9.1% (1)	6.7% (2)	7.3% (3)	0.792
Incomplete LBBB	0% (0)	0% (0)	0% (0)	NA
Ventricular pre-excitation	0% (0)	0% (0)	0% (0)	NA
Pathological Q waves	0.0% (0)	10.0% (3)	7.3% (3)	0.276
>2 PVC per 10 seconds	0% (0)	0% (0)	0% (0)	NA

Ultrasound Measurement Data				

Inner left ventricular diameter (cm)	4.9	5.2	5.1	0.219
Interventricular septal wall thickness (cm)	0.8	1.0	0.9	0.002
Posterior wall thickness (cm)	0.8	1.1	1.0	<0.001
Aortic root breadth (cm)^*^	2.3	2.7	2.6	0.002

*HR*, heart rate; *bpm*, beats per minute; *ms*, milliseconds; *LBBB*, left bundle branch block; *RBBB*, right bundle branch block; *PVC*, premature ventricular contractions; *cm*, centimeters.
