# Supplementary material for: Evaluation of a Standardized Cardiac Athletic Screening for National Collegiate Athletic Association (NCAA) Athletes
Source: West J Emerg Med. 2019 Aug 14;20(5):810–7. doi: 10.5811/westjem.2019.7.43190 (PMC6754195; doi:10.5811/westjem.2019.7.43190)
Supplement: Supplementary file 1 [file wjem-20-810-s001.docx]

**The 14-Element Cardiovascular Screening Checklist for Congenital and Genetic Heart Disease:**

**Personal history:**

1. Chest pain/discomfort/tightness/pressure related to exertion
2. Unexplained syncope/near-syncope*
3. Excessive exertional and unexplained dyspnea/fatigue or palpitations, associated with exercise
4. Prior recognition of a heart murmur
5. Elevated systemic blood pressure
6. Prior restriction from participation in sports
7. Prior testing for the heart, ordered by a physician

**Family history:**

1. Premature death (sudden and unexpected, or otherwise) before age 50 attributable to heart disease in ≥1 relative
2. Disability from heart disease in close relative <50 y of age
3. Hypertrophic or dilated cardiomyopathy, long-QT syndrome, or other ion channelopathies, Marfan syndrome, or clinically significant arrhythmias; specific knowledge of certain cardiac conditions in family members

**Physical examination:**

1. Heart murmur**
2. Femoral pulses to exclude aortic coarctation
3. Physical stigmata of Marfan syndrome
4. Brachial artery blood pressure (sitting position)***

*Judged not to be of neurocardiogenic (vasovagal) origin; of particular concern when occurring during or after physical exertion.
**Refers to heart murmurs judged likely to be organic and unlikely to be innocent; auscultation should be performed with the patient in both the supine and standing positions (or with Valsalva maneuver), specifically to identify murmurs of dynamic left ventricular outflow tract obstruction.
***Preferably taken in both arms.
